# Innovative solutions for PFAS detection under global warming: application prospects of whole-cell bioreporter

**DOI:** 10.3389/fmicb.2025.1682831

**Published:** 2025-09-17

**Authors:** Tao Gan, Williamson Gustave, Boling Li, Christina Lopez, Xiaokai Zhang

**Affiliations:** ^1^School of Environment and Ecology, Institute of Environmental Processes and Pollution Control, Jiangnan University, Wuxi, China; ^2^School of Chemistry, Environmental and Life Sciences, University of the Bahamas, Nassau, Bahamas; ^3^School of Environmental Science and Engineering, Suzhou University of Science and Technology, Suzhou, China; ^4^Meadows Center for Water and the Environment, Texas State University, San Marcos, TX, United States

**Keywords:** global warming, emerging contaminants, biosensor, bioavailability, environmental risk assessment

## Introduction

Perfluoroalkyl And Polyfluoroalkyl Substances (PFAS) are a large group of synthetic chemicals widely used since the 1940's due to their water- and oil-repellent properties (Wang et al., 2017). More than five thousand PFAS compounds have been produced and are commonly found in industrial processes and consumer products (Pelch et al., 2019). Their unique carbon-fluorine bonds confer exceptional chemical stability, making them resistant to natural degradation. As a result, PFAS persist and accumulate in different environmental media globally, posing long-term environmental and toxicological risks (Wang et al., 2025; Young et al., 2021).

Global warming has become a typical feature of climate change. It significantly disrupts environmental systems by altering water flow patterns, sediment transport, and key physicochemical conditions of surface water. Climate-induced changes, such as rising surface water temperatures, increased rainfall intensity, and permafrost thaw, can mobilize legacy PFAS from soils and sediments into aquatic systems. This process broadens the spatial distribution of PFAS and heightens exposure risks to humans and other organisms (Du et al., 2022; Xu et al., 2024). (Shen et al. 2023) demonstrated that increased temperature and humidity weaken the adsorption of PFAS in soil, thereby facilitating their desorption from soil particles and enhancing their bioavailability in terrestrial environments. Meanwhile, alterations in the hydrological cycle can drive the migration of PFAS from sediments to surface water and groundwater, leading to cross-media transport and transformation (Wang et al., 2023; Guo et al., 2025). In addition, elevated temperatures and altered redox conditions under global warming may affect PFAS degradation kinetics, potentially leading to the formation of novel or unknown transformation products with distinct toxicological profiles (Franco et al., 2020). These changes further complicate the transport, transformation, and risk assessment of PFAS in the environment (Gander, 2022). Traditional PFAS detection methods primarily rely on chromatographic–mass spectrometry technologies (e.g., LC-MS/MS), which require cumbersome pretreatment procedures (such as solid-phase extraction), involve high costs, and are time-consuming. Moreover, they can detect only known PFAS congeners and cannot enable real-time monitoring or distinguish between different forms (e.g., bound and free states) (Rodriguez et al., 2020). Furthermore, these methods are prone to background interference from

fluorine-containing instrument components. Moreover, under the combined effects of multiple complex environmental factors in the context of global warming, they are often insufficient to meet the demand for real-time PFAS detection (Menger et al., 2021; Medina and Farmer, 2024). Critically, they cannot provide information on biological impact, limiting their utility in real-time environmental risk assessment.

**Figure 1:**
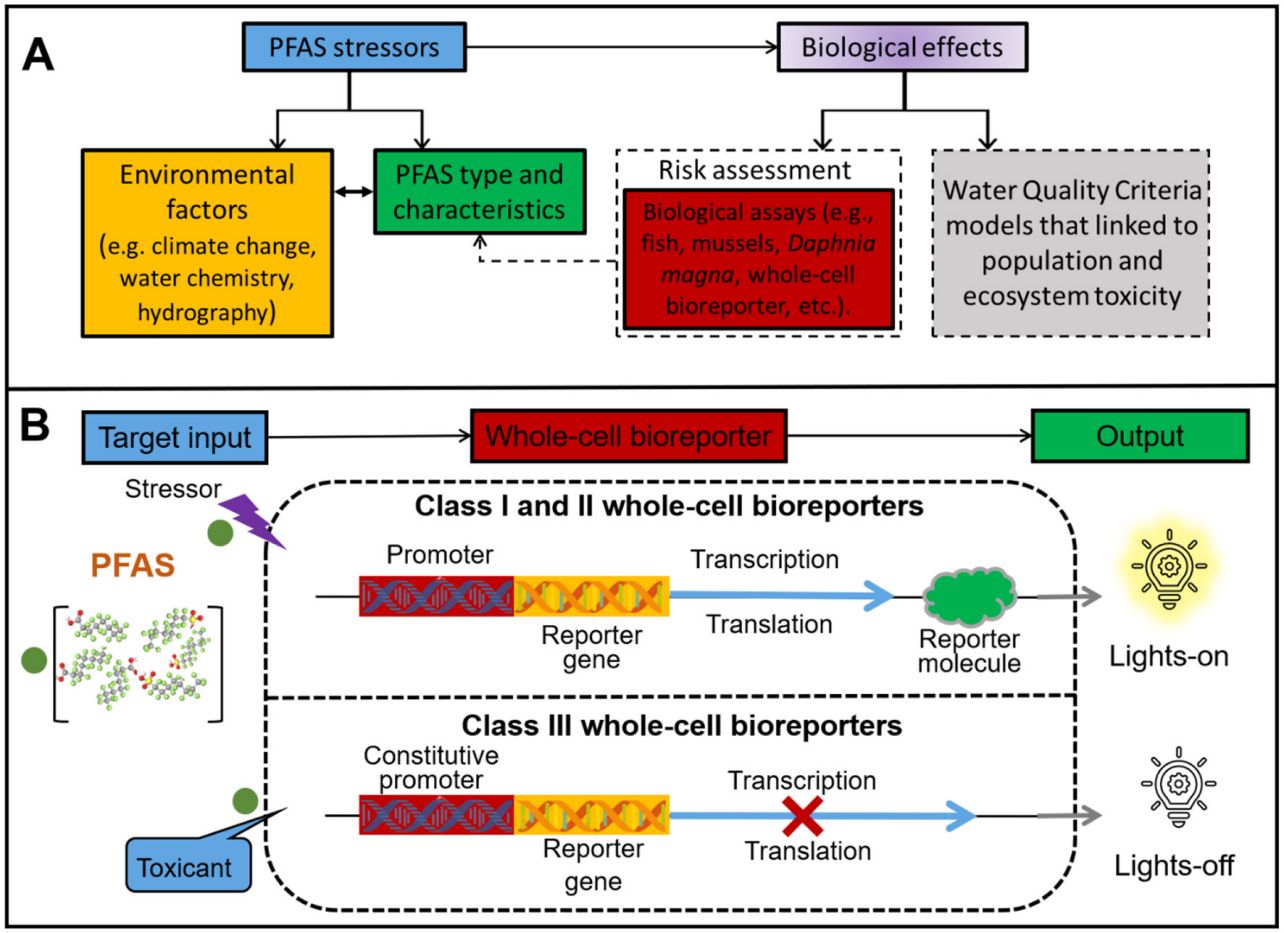
**(A, B)** Schematic A illustrates the conceptual basis of integrated monitoring for PFAS biological effects. Schematic B illustrates the working mechanisms of three classes of whole-cell bioreporters. Class I bioreporters regulate cellular machinery through specific DNA sequences, endowing cells with certain resistance upon exposure to target pollutants. This type of bioreporter exhibits high selectivity when in contact with pollutants. Class II bioreporters are stress-specific whole-cell reporters that exhibit signal output upon sensing stress stimuli (e.g., DNA damage, protein damage, heat shock). This type of reporters cannot distinguish which pollutants cause environmental stress, thus enabling its application in detecting environmental risks induced by multiple pollution. Class III bioreporters are based on constitutively active components of cellular machinery, which drive continuous expression of reporter molecules under normal circumstances; when toxicants disrupt normal cellular processes, the signal diminishes.

A central aspect of PFAS risk lies in their bioavailability, which determines their ecological toxicity. Assessing PFAS bioavailability requires translating chemical exposure into biological responses, often using model organisms (Figure 1A). Traditionally, fish, mussels, and *Daphnia magna* are commonly used model organisms. However, the cultivation and operation of these model organisms are complex and typically require professional technical means. To address these challenges, Whole-Cell Bioreporters (WCBs) have emerged as a promising tool for evaluating pollutant bioavailability (Mann and Berger, 2023). WCBs are genetically engineered living organisms that combine chemical stress with biological impacts (Li et al., 2022). They can sense target chemicals and generate detectable electrochemical or optical signals, thereby determining the bioavailability or toxicity of pollutants (Huang et al., 2024). This technology can circumvent the limitations of traditional methods and is expected to achieve rapid, low-cost, and functional detection in complex environments caused by global warming, providing a particularly suitable tool for the environmental risk assessment of PFAS.

## Research progress on PFAS detection based on WCBs

As shown in Figure 1B, WCBs are categorized into three classes. Class I (“lights-on”) produces dose-dependent signals based on pollutant bioavailability (e.g., zntA for Pb, Li et al., 2022). Class II (“lights-on”) responds to molecular or biological stress (Hartono et al., 2018). Class III (“lights-off”) shows reduced signal due to toxicity, indirectly reflecting bioavailability (Zhu et al., 2022). In recent years, WCBs have gained increasing attention for their potential in detecting PFAS in environmental systems. This technology primarily employs genetically engineered microorganisms (e.g., *Escherichia coli, Alcaligenes eutrophus, Cupriavidus metallidurans, Bacillus subtilis, Staphylococcus aureus*) coupled with fluorescence, bioluminescence, or electrochemical reporting systems to achieve rapid and specific PFAS detection. The advances in PFAS whole-cell bioreporters demonstrate distinct advantages and limitations across different design strategies. (Sunantha and Vasudevan 2021) developed a bioreporter utilizing *Pseudomonas aeruginosa* PAO1, which possesses a complete PFAS metabolic pathway enabling efficient defluorination. By fusing the *pfc-*DEF promoter with the green fluorescent protein (GFP) gene, they innovatively established a sensing system regulated by defluorinating enzyme (*pfc*-DEF) activity. This design converts PFAS degradation by *pfc*-DEF into quantifiable fluorescence signals upon GFP induction. Similarly, *Rhodococcus jostii* RHA1 was engineered as a PFAS bioreporter, leveraging its native stress response mechanism involving *prmA* gene activation upon PFAS exposure. The *prmA* promoter-driven expression of red fluorescent protein demonstrated stable fluorescence output, albeit with a relatively high detection limit (100 μM), highlighting the potential of diverse microbial hosts (Young et al., 2021). These studies successfully converted the stress signals of PFAS into fluorescent signals, quantifying the environmental risks of PFAS. However, bioreporters constructed using these two approaches generally suffer from either prolonged detection times or high detection limits.

In 2023, Mann and Berger constructed a fluorescence-based bioreporter incorporating Human Liver Fatty Acid-Binding Protein (hLFABP), which exhibits moderate affinity for perfluorooctanoic acid (PFOA). By integrating circularly permuted GFP with split hLFABP in *E. coli*, they achieved PFOA detection with limits of 236 μg/L in phosphate buffer and 330 μg/L in environmental water samples, validating the feasibility of whole-cell sensing (Mann and Berger, 2023). Subsequent work in 2024 extended this system to field applications, enabling rapid PFOA screening in industrial wastewater and landfill leachate without extensive pretreatment (Mann et al., 2024). Although the bioreporters developed in these studies exhibit lower detection limits for PFAS compared to previous research, organic matter and other coexisting pollutants in environmental samples may still interfere with the accurate detection of PFAS.

Collectively, these studies highlight several critical innovations in WCB design for PFAS detection: the exploitation of native metabolic or stress pathways, integration of synthetic recognition modules, and expansion of detection formats from fluorescence to electrochemical signals. The described PFAS-detecting WCBs predominantly utilize two types of systems. Class I systems generate dose-dependent signals through direct PFAS recognition, while Class II systems respond to PFAS-induced stress. To date, there are no reported Class III applications for PFAS monitoring. In addition, most systems remain at the proof-of-concept stage, with limited deployment under complex environmental conditions. As global warming continues to increase the variability and unpredictability of PFAS behavior in the environment, the sensitivity and specificity of WCBs must be further enhanced for real-world application.

## Discussion

As discussed above, global climate change has led to greater complexity in the environmental behaviors of PFAS in multiple media (Wang et al., 2023). These evolving conditions have exposed the limitations of conventional chemical analytical methods and underscore the need for more adaptive, biologically relevant approaches. WCB technology has demonstrated unique advantages in environmental monitoring after nearly three decades of development. However, most current WCB research primarily focuses on assessing the bioavailability of heavy metals and typical organic pollutants (e.g., polycyclic aromatic hydrocarbons). In contrast, emerging pollutants, such as PFAS, bioavailability detection remains relatively underexplored. Furthermore, investigations have revealed that the few existing WCBs for PFAS detection are all focused on aquatic environments. However, significant challenges remain in the detection and risk assessment of PFAS in multiple media, such as soil and sediment. Components like soil particles and humic substances may interfere with bioreporter signals, thereby reducing detection sensitivity and compromising the accuracy of detection results (Kolosova et al., 2022).

A major barrier to practical implementations also lies in ensuring adequate specificity and sensitivity (Bhatt et al., 2024). Natural environments often contain complex mixtures of PFAS compounds with structural similarities (Chen et al., 2017), complicating the discrimination between target analytes and interfering substances. Furthermore, temperature fluctuations, pH variations, and co-existing contaminants (e.g., heavy metals, pesticides) induced by climate warming may impair host cell viability and signal stability (Liu et al., 2022; Cai et al., 2023). Future advancements should integrate biological tools with novel recognition elements to enhance performance, alongside engineering stress-resistant microbial chassis and immobilization techniques to improve environmental adaptability. Furthermore, to accelerate the transition from laboratory research to practical implementation, it is crucial to establish standardized evaluation protocols and optimize bioreporter performance under environmentally relevant conditions. Future research should focus on improving strain robustness, developing multiplexed detection capabilities, and validating long-term stability across diverse aquatic environments. Collaborative efforts between academia, industry, and regulatory bodies will be essential to address current technical limitations and facilitate the integration of WCB technology into routine environmental monitoring programs for PFAS contamination.
